# A Differential Effect of *E. coli* Toxin-Antitoxin Systems on Cell Death in Liquid Media and Biofilm Formation

**DOI:** 10.1371/journal.pone.0006785

**Published:** 2009-08-26

**Authors:** Ilana Kolodkin-Gal, Reut Verdiger, Ayalla Shlosberg-Fedida, Hanna Engelberg-Kulka

**Affiliations:** Department of Molecular Biology, Hadassah Medical School, The Hebrew University, Jerusalem, Israel; Charité-Universitätsmedizin Berlin, Germany

## Abstract

Toxin-antitoxin (TA) modules are gene pairs specifying for a toxin and its antitoxin and are found on the chromosomes of many bacteria including pathogens. Here we report how each of five such TA systems in *E. coli* affect bacterial cell death differently in liquid media and during biofilm formation. Of all these systems, only the TA system *mazEF* mediated cell death both in liquid media and during biofilm formation. At the other extreme, as our results have revealed here, the TA system *dinJ-YafQ* is unique in that it is involved only in the death process during biofilm formation. Cell death governed by *mazEF* and *dinJ-YafQ* seems to participate in biofilm formation through a novel mechanism.

## Introduction

Toxin-antitoxin systems consist of a pair of genes that specify two components: a stable toxin and an unstable antitoxin that interferes with the action of the toxin. Several toxin-antitoxin modules have been identified in the chromosome of *E. coli*. Among them are: *mazEF* [1]–[3], *chpBIK* [1], [4], *relBE* [5]–[7], *yefM-yoeB* [8]–[10], *dinJ-yafQ* [11]. The well studied *mazEF* system was the first to be described as regulatable and responsible for bacterial programmed cell death [12]. *mazF* encodes for the stable toxin MazF and *mazE* encodes for the labile antitoxin MazE. MazE is degraded by the ATP-dependent ClpPA serine protease[12]. MazF is an endoribonuclease that cleaves mRNAs at ACA sequences in a ribosome-independent manner [13], [14]. As long as MazE and MazF are co-expressed, MazE counteracts the toxic activity of MazF [12]. Since MazE is a labile protein, preventing MazF-mediated action requires the continuous production of MazE. Thus, any stressful condition that prevents the expression of the chromosomally borne *mazEF* module will lead to the reduction of MazE in the cell, permitting toxin MazF to act freely. Such conditions include: (i) antibiotics inhibiting transcription and/or translation like rifampicin, chloramphenicol, and spectinomycin [15]; and ii) antibiotics causing DNA damage like trimethiprim or nalidixic acid [16], [17]. These antibiotics, and some other stressful conditions that are well known to cause bacterial cell death [18], [19], have been found to act through the *mazEF* module [15]–[17].

Another, well studied TA system is *relBE*, which is induced by stressful conditions that cause growth arrest by allowing the toxin RelE to act freely [20]. RelE causes cleavage of mRNA codons in the ribosomal A site. This cleavage is highly codon specific, occurring between the second and third nucleotides [21]. It has been suggested that RelE might not be an endonuclease itself, but rather may enhance the intrinsic cutting action of the ribosome when it pauses in the translation process [22]. The antitoxin RelB is degraded by the Lon protease [20].

Other than *mazEF* [23] and *relBE* [6], [7], [20], most other *E. coli* TA systems have been discovered only recently. Each of them inhibits translation; however, the various toxins differ in their primary targets and modes of action. *chpBIK* is partially homologous to *mazEF* [1]. Like MazF, the toxin ChpK is also an endoribonuclease that cleaves mRNAs in a ribosome-independent manner [14]. Unlike MazF that cleaves mRNAs only at ACA sequences [13], ChpK also cleaves at sequences ACU and ACG [14]. YoeB was also initially thought to function as an endoribonuclease cleaving translated mRNAs [9]. However, recently YoeB was found to be specifically associated with the 50S ribosomal subunit of *E. coli* and thereby it primarily inhibits translation initiation [24]. YafQ of the *dinJ-yafQ* system has been recently shown to be a toxin that functions different from other TA toxins. It is an endoribonuclease that associates with the ribosome and blocks translation elongation through sequence–specific and frame dependent mRNA cleavage [25]

Because of the similar organization of the genes in the TA modules, and the similar concept of specifying a stable toxin and labile antitoxin, TA modules are generally viewed as having similar roles in physiological processes. However, based on the fact that the mode of action the studied *E. coli* toxins varies, they may also differ in their role in bacterial physiology. Therefore, here, using similar experimental conditions, we compared the effects on cell death of the five confirmed *E. coli* TA systems (*mazEF*, *chpBIK*, *relBE, yefM-yoeB*, and *dinJ-yafQ*) in liquid media and in bioifilm formation. We found that these TA systems can be divided into four groups: 1) *mazEF* is involved in the death processes in both liquid and biofilm formation; 2) *relBE* only participates in cell death in liquid medium; 3) *chpBIK* and *yefM-yoeB* only participate in cell death in liquid medium under certain conditions; and 4) *dinJ-yafQ* only participates in the death process involved in biofilm formation, but not in liquid medium. We have shown, for the first time, that the process of *E. coli* biofilm formation required cell death which is governed by the TA systems *mazEF* and *dinJ-yafQ*. We particularly found that the *dinJ-yafQ* gene pair is a unique TA system that participates in the cell death process only in biofilm formation.

## Results

### Overproduction of each of five chromosomal *E. coli* toxins has a different effect on cell viability in liquid media

**Figure 1:**
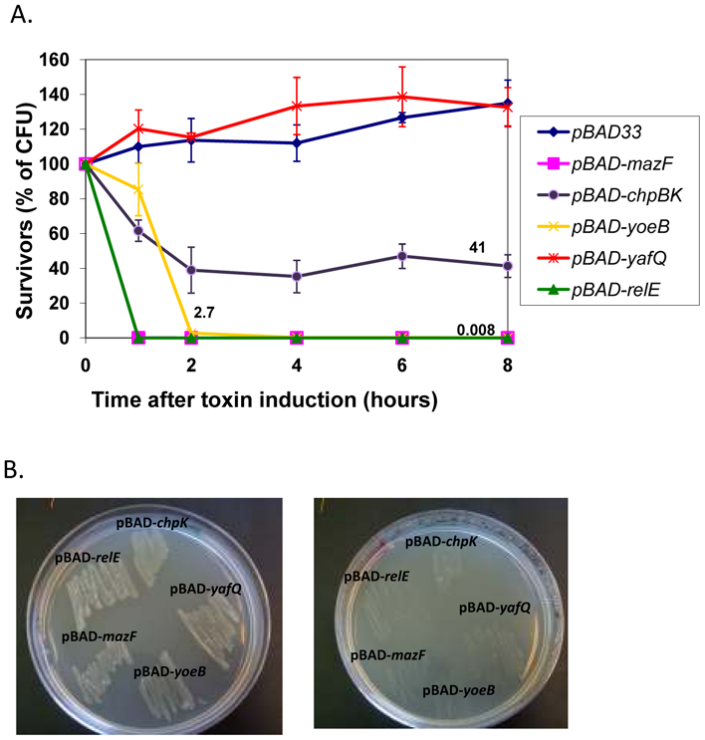
The effect of the overproduction of each of five *E. coli* toxins on *E. coli* survival in liquid (A) and solid media (B). (A) Cells were grown in LB medium at 37°C to mid-logarithmic phase (OD_600_ = 0.5). At time zero, 0.2% arabinose was added to the cultures to induce the toxin expression (Materials and Methods). (B) Overproduction of five of *E. coli* toxins in cells grown on solid medium. *E. coli* strain MC4100 containing BAD33 carrying each of five of the chromosomal *E. coli* toxins were plated on LB medium plates (left) or LB plates applied with 0.2% arabinose (right) and incubated at 37°C.

We wished to compare the toxicity of each of five overproduced *E. coli* chromosomal toxins. To this purpose, we used *E. coli* strain MC4100*relA* ^+^ transformed with plasmid pBAD33 carrying an insert of each of five *E. coli* toxins under the regulation of the *araBAD* promoter. We grew the transformed cells in liquid LB medium to mid-logarithmic phase, and then induced the toxins by adding arabinose. We found that overproducing each of these *E. coli* toxins led to significantly different results (Figure 1A): The most rapid and dramatic loss of viability was caused by overproducing either MazF or RelE: in only one hour, the number of colony forming units was decreased by 3 orders of magnitude, in 6 hours by more than 4 orders of magnitude. Overproducing YoeB caused a similar reduction in cell viability, but only 2 hours after the toxin was induced. Overproducing ChpK affected cell viability only about half as much. Surprisingly, overproducing YafQ did not affect cell viability at all (Figure 1A); even in defined M9 medium, in which overproducing the other four toxins caused drastic reductions in cell viability, overproducing YafQ had no effect (Figure S1). YafQ is a functional toxin, because, as demonstrated previously and here (Figure 1B, Figures S2A and B), when tested for its ability to induce growth arrest, YafQ prevents cell growth as do other E. coli toxins.

### Each of five of chromosomal *E. coli* toxin-antitoxin system has a different effect on cell viability following treatment with various antibiotics

**Figure 2:**
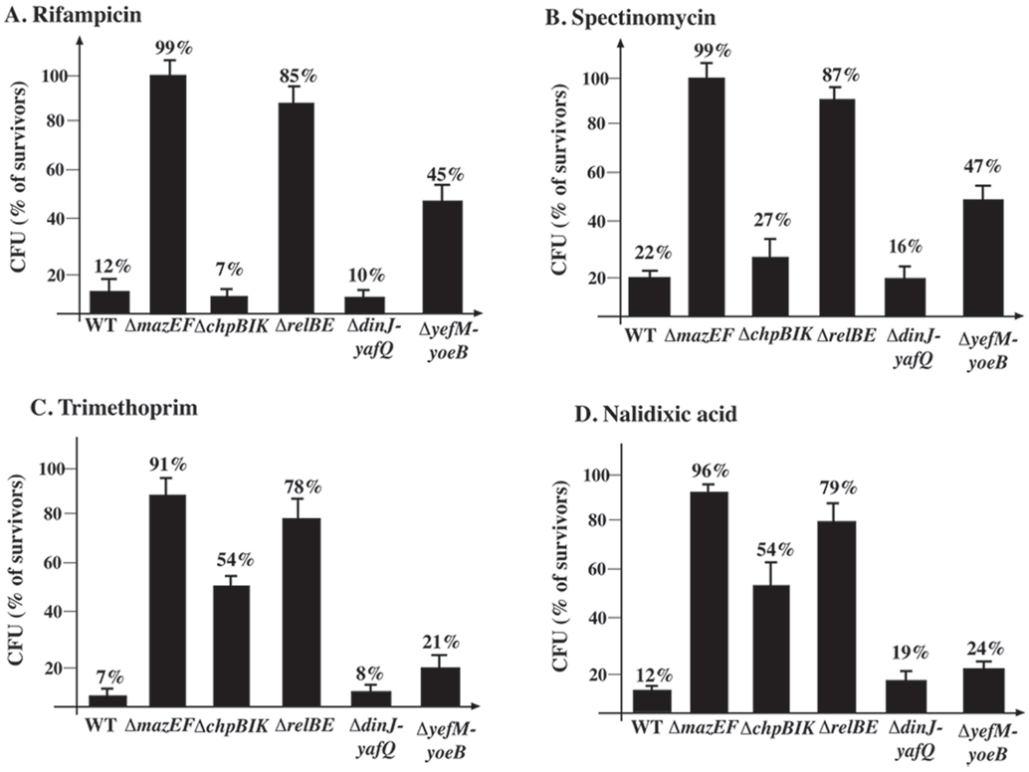
The effect of each of five of chromosomal encoded TA systems on *E. coli* cell survival following treatment with different antibiotics. *E. coli* strains MC4100*relA* ^+^ (WT) and its Δ*mazEF*, Δ*chpBIK*, Δ*relBE*, Δ*yefM-yoeB*, or Δ*dinJ-yafQ* derivatives were grown in M9 medium to mid-log phase (Materials and Methods). Cells were incubated without shaking at 37°C with: (A) Rifampicin (10 µg/ml) for 10 min; (B) Spectinomycin (1 mg/ml) for 10 min; (C) Trimethoprim (2 µg/ml) for 1 hr; (D) Nalidixic acid (1 mg/ml) for 10 min (Materials and Methods). The results describe the average of three independent experiments that were carried out in triplicate. Error bars indicate standard deviations.

Having found previously that *mazEF* mediated cell death is triggered by various antibiotics [15]–[17], we asked if cell death triggered by antibiotics might involve additional TA systems. We compared the viability of wild-type *E. coli* MC4100*relA* ^+^ to the viability of mutants from which each of the five TA gene pairs had been deleted individually. Deleting each of the five TA systems affected cell survival differently (Figure 2). Mutants deleted for either *mazEF* or *relBE* did not die following treatment with various antibiotics (Figure 2). The effect on cell death of deleting *yefM-yoeB* or *chpBIK* was dependent on which antibiotics were used to trigger the cell death process: after treatment with antibiotics inhibiting transcription or translation, we observed only about 50% cell death in the *ΔyefM-yoeB* mutant (Figure 2A,B). In the *ΔchpBIK* mutant, there was a reduction in cell death only after treatment by DNA damaging agents (Figure 2C,D). Deleting *dinJ-yafQ* had no significant effect on cell death regardless of the antibiotic used to trigger the death process (Figure 2).

Since our experiments were carried out in bacterial strains that carried an intact *relA* gene, we also compared the effect on cell death of deletion of each of five TA systems in MC4100*relA1* background. Since, as we have described previously, ppGpp has a role in *mazEF*-mediated cell death [17], using the same experimental conditions as for the *relA* ^+^ strain we were not able to induce cell death in a *relA*1 strain (Data not shown). However, using a more drastic treatment with different antibiotics, we did observe *mazEF* and *relBE*-mediated cell death (Figure S3). We were surprised that in *relA1* background, the Δ*chpBIK* mutant survived all stressful conditions, suggesting that the presence of ppGpp may disguise the involvement of the *chpBIK* TA system in cell death (Figure S3).

These results suggest that *mazEF* and *relBE* are the principal TA systems participating in cell death induced by antibiotics; that *chpBIK* and *yefM-yoeB* are TA systems that only in some cases participate in cell death, and that *dinJ-yafQ* has no role in this cell death process.

### *dinJ-yafQ* and *mazEF* are involved in cell death during biofilm formation

**Figure 3:**
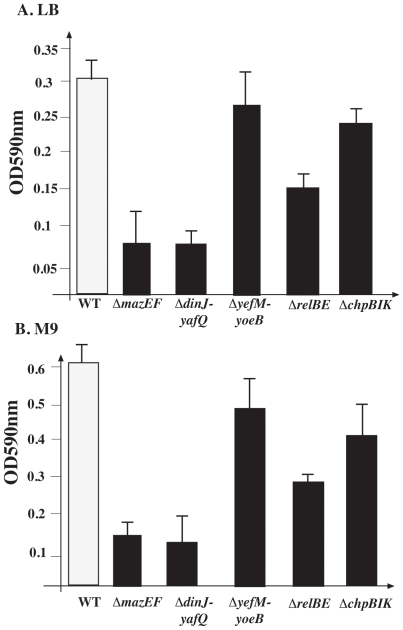
The effect of each of five of chromosomal borne TA systems on *E. coli* biofilm formation. *E. coli* strains MC4100*relA* ^+^ (WT) and its Δ*mazEF*, Δ*chpBIK*, Δ*relBE*, Δ*yefM-yoeB* or Δ*dinJ-yafQ* derivatives were grown in 96 well polystyrene plates at 37°C for 24 hr in (A) LB or (B) M9. Quantification of CV-stained attached cells was done as described in Materials and Methods. The results describe the average of three independent experiments that were carried out in triplicate. Error bars indicate standard deviations.

**Figure 4:**
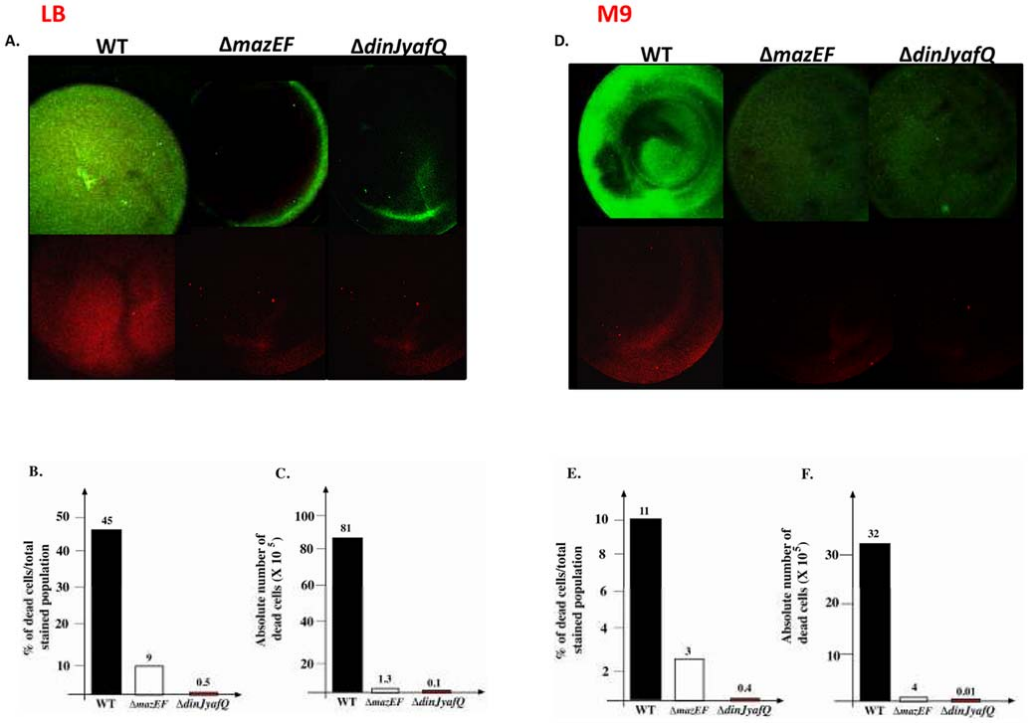
The effect of each of *mazEF* and *dinJ-yafQ* TA systems on *E. coli* cell death during biofilm formation. *E. coli* strains MC4100*relA* ^+^ (WT), MC4100*relA* ^+^Δ*mazEF*, and MC4100*relA* ^+^ Δ*dinJ-yafQ* were grown at 37°C for 24 hr in 96 well polystyrene plates in LB (A,B,C) or M9 (D,E,F) media. Attached cells were washed and planktonic cells were removed as described (Materials and methods). Dead cells stained with PI (red) and living cells retained staining with SYTO 9 (green). (A,D) Biofilms were photographed by CLSM with a using ×2.5, ×10 magnifications. The entire well was photographed from above. The image is a representative image from three independent experiments; each experiment was carried out in quadruplicate. Additionally, cells stained with SYTO 9 or PI were quantified as described in “materials and methods”. (B,E) Percentage of dead cells from the total population in the biofilm. (C,F) Absolute number of dead cells. Data were obtained from two independent experiments, performed quadruplicate.

We also asked whether the differential effect of TA systems is also reflected in biofilm formation. Using the *E. coli* deletion mutants described above, we studied the effect of each of the TA systems on biofilm formation. As early as 8 hours, we observed a significant decrease in biofilm formation in both the *ΔmazEF* and the *ΔdinJ-yafQ* mutants (Figure S4). Particularly in a defined medium, we observed only a partial decrease in biofilm formation in the *ΔchpBIK, ΔrelBE*, and *ΔyefM-yoeB* mutants (Figure S4). We subsequently studied the effects on later biofilm formation, after 24 hours, of deleting *mazEF* or *dinJ-yafQ*. Indeed, compared to their parental strain, after 24 hours, biofilm production was significantly reduced in both *ΔmazEF* and *Δdinj-yafQ* mutants (Figure 3 and Figures 4A,D). In rich LB medium, deleting *dinJ-yafQ* or *mazEF* almost completely abolished biofilm formation (Figure 4A, upper panel). In M9 minimal medium, deleting *dinJ-yafQ* or *mazEF* resulted in the formation of very thin, impaired biofilms (Figure 4D, upper panel).

In a bacterial culture, the death of some of the cells may provide extra nutrient, signaling, and extra-cellular matrix molecules for the cells that remain living [27]. It seems likely that biofilm formation would be supported by an increase in the abundance of such molecules. We hypothesized that the affects of deleting *dinJ-yafQ* or *mazEF* on biofilm production could be the result of a defect in a cell death pathway. Based on this assumption, we predicted that biofilms produced by the WT strain would contain a larger proportion of dead cells then those produced by the *ΔmazEF* or the *ΔdinJ-yafQ* mutants. To test this idea, we used propidium iodide (PI) and SYTO 9, to distinguish between living and dead cells. When used alone, SYTO 9 stains both live and dead bacteria. In contrast, PI penetrates only dead bacteria with damaged membranes. When used together, PI causes a reduction in SYTO 9 fluorescence: live bacteria with intact membranes fluoresce green, while dead bacteria with damaged membranes fluoresce red. We analyzed a 24-hr biofilms produced by each strain, and stained them with both PI and SYTO 9, (Figures 4A,D, lower panels). We calculated the percentage of dead cells in each biofilm by dividing the number of dead cells by the total number of cells (dead+live cells). We found that in both LB and in M9 media, the fraction of dead cells in the biofilm of the WT parental strain contained 100 times more dead cells than the biofilm of the *dinJ-yafQ* deletion mutant (Figures 4B and 4E). In LB medium, the biofilm of the *mazEF* deleted mutant contained 10 times fewer dead cells than the biofilm of the parental strain (≈9% versus 45% Figure 4B). Based on these results, we predicted that optimal biofilm formation might require a minimal threshold number of dead cells, so we determined the absolute number of dead cells in each case. Compared to the biofilms of the *ΔmazEF* or the *ΔdinJ-yafQ* mutants, the biofilm of the parental WT strain included at least 2 orders of magnitude more dead cells (Figures 4C and 4F). This suggested that *mazEF* and *dinJ-yafQ* mediated cell death in *E. coli* play a pivotal role in biofilm formation. The requirement for the active cell death process mediated by the TA systems *mazEF* and *dinJyafQ* for proper biofilm formation is also reflected by the significant difference(s) in the number of total viable cells inside the biofilm. The biofilm of both *mazEF* and *dinJyafQ* mutants contain at least 10 times less viable cells than their parental WT strain (data not shown).

Based on the results described above, we reasoned that overexpression of the MazF and YafQ toxins may increase biofilm formation in Δ*mazEF* and Δ*dinJ-yafQ*. After overexpressing MazF and YafQ with 0.05% arabinose, we found that both toxins fully complemented the defect in biofilm formation of their cognate TA deleted mutant (Figure S7). This result further support our finding that the defect of the mutants deleted for either *dinJ-yafQ* or *mazEF* in biofilm formation is a result of a defect in cell death pathway (Figure 4).

Note that for biofilm formation over a 24 hour period, in both in rich LB medium (Figure S5A lower panel and Figures S5B,C), and in a defined M9 defined medium (Figure S6A lower panel and Figures S6B,C) deleting the TA systems *chpBIK*, or *yefM-yoeB* had much less significant effect than deleting either *mazEF* or *dinJ-yafQ*. In addition, though deleting the *relBE* TA gene pair led to a partial defect in biofilm formation (Figures 3, S4, S5A, S6A), the loss of *relBE* had no effect on the proportion of dying cells during 24 h of biofilm formation comparing with the WT strain (Figure S5 and S6). Thus, we suggest that there may be an additional role for the *relBE* TA system in biofilm formation that is not connected to cell death.

## Discussion

It is well known that bacteria can undergo between two physiological states: a free-swimming plaktonic state and in surface-associated communities called biofilms [26]. Biofilms are viewed as complex communities of bacteria resulting through multi-developmental stages that can be viewed as a multicellular behavior [26]. We have previously described the *E. coli* TA module *mazEF* programmed cell death system as one of the facets of multi-cellular behavior in bacterial populations. When challenged by stressful conditions, the bacterial population acts like a single multicellular organism in which a sub-population dies, thereby permitting the continued survival of the bacterial population as a whole [28], [29]. So, here we asked: Is *mazE*F mediated cell death involved in biofilm formation? Moreover, if *mazEF* is involved, is it a representative of other *E. coli* TA systems?

**Figure 5:**
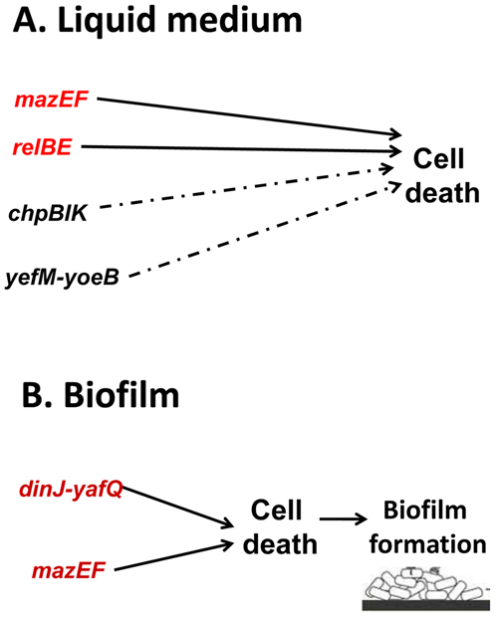
A model of the effect of each of five chromosomal borne TA systems on *E. coli* cell death in liquid media and during biofilm formation. Principle TA systems for cell death are in red, and secondary in black broken arrow.

We found that with respect to cell death, the several TA systems behaved differentially (Figure 5). Based on our previous studies [23], [29] and here, *mazEF* is the regulating module mediating cell death both in liquid media (Figure 2) and in biofilm formation (Figures 3 and 4). *relBE* seems to be a principal mediator of cell death only in liquid media (Figure 2, [30]), but not in biofilm formation (Figure 3). We found that *chpBIK*, which is homologous to *mazEF* [1], [4], seems to be a back-up death system for *mazEF*. In a *relA* ^+^ *E. coli* strain in which ppGpp is produced [31], the principal death mediator was *mazEF* (Figure 2); however, in the isogenic *relA1* strain, in which ppGpp is not produced [31], *chpBIK* was required to mediate cell death (Figure S3). In addition, under our experimental conditions, the *yefM-yoeB* module mediates cell death only in liquid media, but only in some cases (Figure 2, and Figure S3), while not at all in bioiflm formation (Figure 3, Figure S5 and S6). The results of this study revealed that that the TA system *dinJ-yafQ* is unique: even when the toxin YafQ is over-expressed, it may not mediate cell death in liquid media (Figures 1 and 2). However, strikingly, *dinJ-yafQ* seems to be a principal mediator of cell death in biofilm formation (Figures 3 and 4). This is the first study showing a specific function for *dinJ-yafQ.*

It was recently reported that all *E. coli* TA systems are involved in biofilm formation [32]. However, these experiments were performed in strain MG1655, which is defective in cell death [33]. We report here the following novel findings regarding *E. coli* TA systems and biofilm formation: (i) Among the five studied *E. coli* TA systems, only *mazEF* and *dinJ-yafQ* have a role; (ii) The role of *mazEF* and *dinJ-yafQ* in biofilm formation is related to their role cell death; and (iii) A threshold of dead cells seems to be important for biofilm formation. We are presently studying the specific stage in biofilm formation that may be affected by *mazEF* and/or *dinJ-yafQ* mediated cell death and whether these may be related to the planktonic death pathways about which we have reported [28], [29]. It was previously reported that *cidA-*controlled cell lysis has a significant role in biofilm development in *Staphylococus aureus*, and that the released genomic DNA is an important structural component of the biofilm of this bacteria [34]. In our work here, we found that the addition of DNAse did not reduce *E.coli* biofilm formation (Figures S8 and S9). It seems, therefore, that the role of cell death in *E.coli* biofilm formation is not through the release of DNA. Thus, *mazEF* and *dinJ-yafQ* mediated cell death participate in biofilm formation through novel yet unknown mechanism(s).

## Materials and Methods

### Bacterial strains and plasmids

*E. coli* strains: MC4100*relA^+^*, its Δ*mazEF* derivative [26], MC4100*relA1* and its Δ*mazEF* derivative [17]. Using Red-mediated homologous recombination [35], we constructed the chloramphenicol resistant derivatives: Δ*relBE*, Δ*dinJ-yafQ*, Δ*chpBIK* and Δ*yefM-yoeB* of *E. coli* strains MC4100*relA^+^* and MC4100*relA1*. For strain MC4100*relA^+^* Δ*dinJ-yafQ*, the Cam^R^ was eliminated [35]. We used pBAD-*mazF* [36], and constructed pBAD-*yafQ* as follows: *yafQ* gene was PCR amplified from strain MC4100 and cloned using KpnI and HindIII sites into the plasmid pBAD33 [37] bearing an chloramphenicol resistance gene, downstream of the arabinose pBAD promoter.

### Materials and media

The bacteria were grown in liquid M9 defined medium [27] with 1% glucose and applied with each amino acid (10 µg/ml) or LB [17] and then plated on rich LB agar plates [17]. The following materials were obtained from Sigma: L-arabinose, nalidixic acid, mitomycin C, trimethoprim, rifampin, serine hydroxamate, chloramphenicol, spectinomycin, crystal violet and DNAse I. PI and SYTO 9 were obtained from Invitrogen (Carlsbad, California).

### Determining the effect of each toxin overproduction

*E.coli* strains MC4100 deleted with each of the five TA modules were transformed with pBAD33 carrying an insert of its cognate toxin and were grown in M9 medium containing 0.5% glycerol as a carbon source or in LB medium with chloramphenicol (50 µg/ml). Then cells were treated as described in Figure 1 and S1 legends. To determine CFU, samples were withdrawn at various time points and spread on LB plates containing 50 µg of chloramphenicol per ml and 0.2% glucose to repress further expression of the toxins. The percentage of the survivors was calculated by comparing the CFU of the induced culture to that of the uninduced culture at time zero. Error bars indicate standard deviations.

### Determining the effect of stressful conditions on *mazEF*-mediated cell death

Cells were grown in M9 medium with shaking (160 rpm) at 37°C for 12 hours. The cells were diluted 1∶100 in 10 ml of M9 medium and were grown with shaking (160 rpm) at 37°C to mid-logarithmic phase (OD_600_ 0.6). Samples of 500 µl were withdrawn into Eppendorf tubes (1.5 ml volume) and were further incubated without shaking at 37°C for 10 min as described for each case. Antibiotics were applied as described in each figure legend. The cells were centrifuged and re-suspended in pre-warmed saline, diluted, plated on pre-warmed LB plates and incubated at 37°C for 12 hours. Cell survival was calculated by comparing the number of the colony-forming units of cells treated by stressful conditions to those of the cells that were not exposed to the treatment.

### Quantification of Crystal Violet (CV) -stained attached cells

Cells were grown overnight in LB or M9 medium. Then, cells were diluted 1∶100 in the same medium and grown in standing 96 wells polystyrene (without shaking). Quantification of CV stained attached cells was done as described previously (38) with a few modifications: After the wells were stained with 125 µl of 1.0% CV, rinsed and thoroughly dried, the CV was solubilized by the addition of 200 µl of 95% ethanol. A 125 µl sample of the solubilized CV was removed and added to a fresh polystyrene 96-well dish, and absorbance at OD_590_ was determined using Microplate Reader from from BMC (Ontario, Canada).

### Determination of the Number of Dead cells in *E. coli* biofilms

*E.coli* cells were grown in 96 wells polystyrene plates as described in Figures 4, S5 and S6 legends. The culture supernatants were discarded, and the leftover biofilms in each well were fixed in 3% formalin solution for 15 min. The supernatants were discarded, and the leftover biofilms were washed with saline. Biofilms were incubated with a solution of 4 mM PI and 4 mM SYTO 9 for additional 15 min. The wells were washed again in saline and applied with 100 µl of 50% glycerol. Wells were analyzed by confocal laser scanning microscopy (CLSM) using a LSM 410 confocal microscope (Carl Zeiss, Inc., Thornwood, NY) under 1,000 magnification. Image acquisition was performed by using Carl Zeiss LSM software version 3.99 (Carl Zeiss, Inc.). After counting all six fields of view, the number of red cells was divided by the total number of cells (red+green) and multiplied by 100 to calculate the percentage of dead cells in each sample.

## Supporting Information

Figure S1 The effects of overproducing five *E. coli* toxins in cells grown in liquid M9 minimal medium. The *E. coli* strains used in the experiments from Fig. 1 were treated as described in the Legend to Fig. 1 except that cells were grown in M9-Glycerol medium (rather than in LB).
(0.04 MB PDF)

Figure S2 Growth arrest induced by the overproduction of five of *E. coli* chromosomally borne toxins. *E. coli* strain MC4100 was transformed with pBAD33 carrying an insert of one of five *E. coli* toxins. Cells were grown at 37°C in (A) liquid LB medium with 0.2% glucose (B) glycerol M9 minimal medium to an A600nm = 0.3–0.5. Then, growth medium of was changed into fresh medium with 0.2% arabinose. Bacterial growth was assessed by measuring A600 nm every 30 min.
(0.05 MB PDF)

Figure S3 The effect of each of five of chromosomal encoded TA systems on E. coli cell survival following treatment with different antibiotics. *E. coli* strains: MC4100*relA1* (WT) and its D*chpBIK*, D*relBE*, D*yefM-yoeB* and D*dinJ-yafQ* derivatives (in gray) were grown to mid-log phase as described in Materials and Methods. Cells were incubated without shaking at 37°C with: (A) Rifampicin (25 µg/ml) for 10 min; (B) Spectinomycin (2 mg/ml) for 10 min; (C) Trimethoprim (2 µg/ml) for 2 hr; (D) Nalidixic acid (2 mg/ml) for 10 min. Cells were plated and CFUs assessed as described in Materials and Methods.
(0.29 MB PDF)

Figure S4 The effect of each of five of chromosomal encoded TA systems on early biofilm formation in *E. coli*. *E. coli* strains *MC4100relA+* (WT) and its D*mazEF*, D*chpBIK*, D*relBE*, D*yefM-yoeB* or D*dinJ-yafQ* derivatives were grown in 96 well polystyrene plates at 37°C for 8 hr in (A) LB or (B) M9. Quantification of CV-stained attached cells was done as described in Materials and Methods.
(0.18 MB PDF)

Figure S5 The effects of D*chpBIK*, D*relBE* and D*yefM-yoeB* on *E. coli* cell death during biofilm formation in LB medium. *E. coli* strains: MC4100*relA+* (WT) and its derivatives D*chpBIK*, D*relBE*, and *yefM-yoeB* were grown in 96 wells polystyrene plates at 37°C for 24 hr in LB medium. For the rest of the experiment, see the Legend to Fig. 4.
(6.81 MB PDF)

Figure S6 The effect of the deletions D*chpBIK*, D*relBE* and D*yefM-yoeB* on *E. coli* cell death during biofilm formation in M9 medium. *E. coli* strains MC4100*relA+* (WT) and its D*chpBIK*, D*relBE*, and D*yefM-yoeB* derivatives were grown in 96 well polystyrene plates at 37°C for 24 hr in M9 medium. For the rest of the experiment, see the Legend to Fig. 4.
(5.07 MB PDF)

Figure S7 Overproduction of YafQ and MazF restores biofilm formation in a D*mazEF* and D*dinJ-yafQ* derivatives. *E. coli* strains MC4100*relA+* (WT), MC4100*relA+DmazEF*/pBAD-*mazF* and MC4100*relA+DdinJ-yafQ*/pBAD-*yafQ* were grown in 96 wells polystyrene plates at 37°C in LB or LB+Arabinose 0.05% for (A) 8 h (B) 24 h. Quantification of CV-stained attached cells was done (Materials and Methods).
(0.20 MB PDF)

Figure S8 DNAse treatment does not affect early biofilm formation in *E. coli*. *E. coli* strains MC4100*relA+* (WT) or its D*mazEF* and D*dinJ-yafQ* derivatives were grown in 96 wells polystyrene plates at 37°C for 8 h in (A)LB (B) M9. DNAse (1 or 10 Kunitz) was either applied or not applied to each well. Quantification of CV-stained attached cells was done (Materials and Methods).
(0.21 MB PDF)

Figure S9 *E. coli* strains MC4100*relA+* (WT) or its D*mazEF* and D*dinJ-yafQ* derivatives were grown in 96 wells polystyrene plates at 37°C for 24 h in (A)LB (B) M9. DNAse (1 or 10 Kunitz) was either applied or not applied to each well. Quantification of CV-stained attached cells was done (Materials and Methods).
(0.22 MB PDF)
