# Correction: A Differential Effect of *E. coli* Toxin-Antitoxin Systems on Cell Death in Liquid Media and Biofilm Formation

**DOI:** 10.1371/journal.pone.0140184

**Published:** 2015-10-02

**Authors:** Ilana Kolodkin-Gal, Reut Verdiger, Ayalla Shlosberg-Fedida, Hanna Engelberg-Kulka

There are errors in the lower panels of Fig 4A and Fig 4D of the published article. Please see the correct Fig 4 here. The original images for all of the lower panels in Fig 4 are included as Supporting Information files to this correction. The authors apologize for the errors.

**Fig 4:**
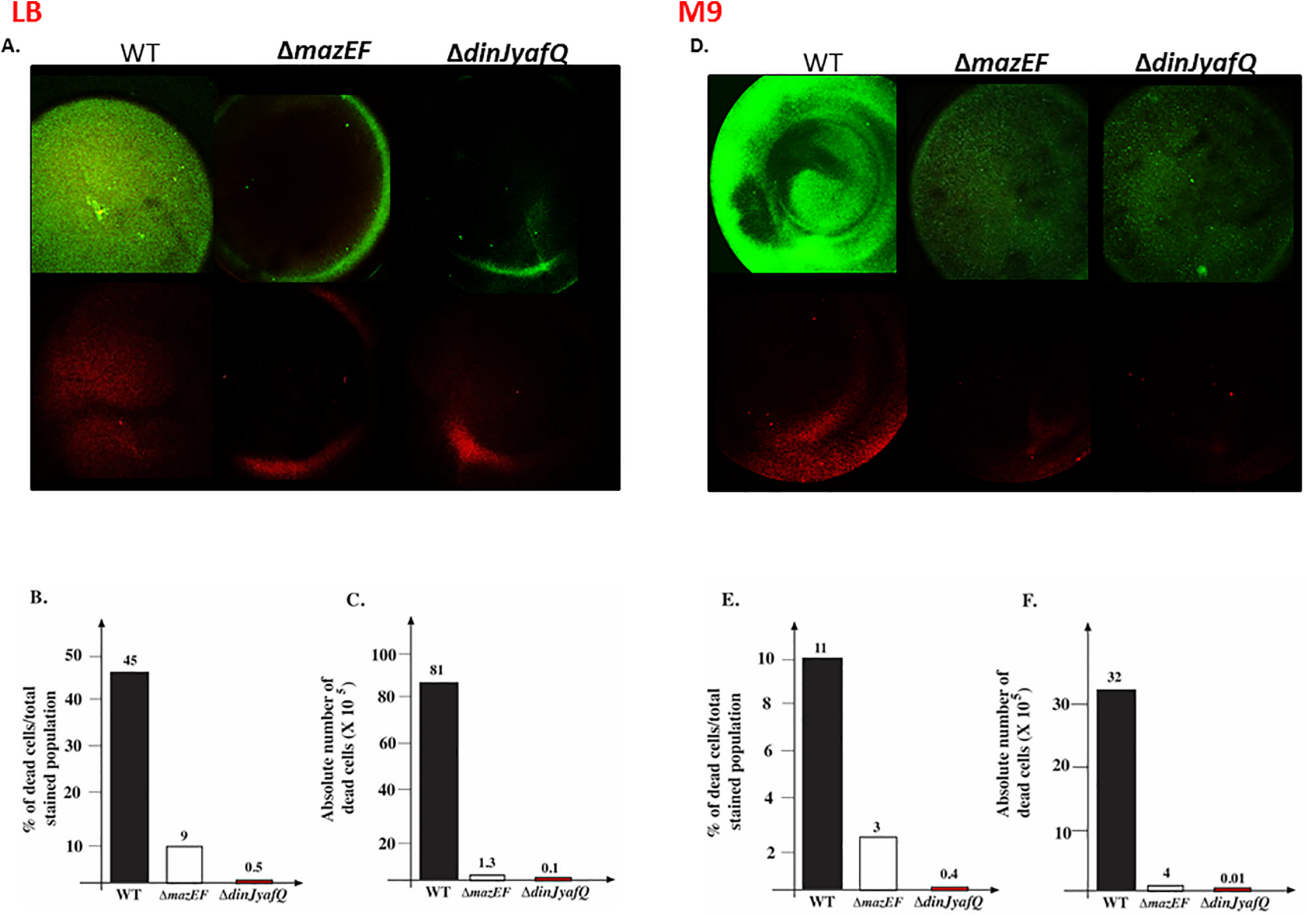
The effect of each of *mazEF* and *dinJ-yafQ* TA systems on *E*. *coli* cell death during biofilm formation. *E*. *coli* strains MC4100*relA* ^+^ (WT), MC4100*relA* ^+^Δ*mazEF*, and MC4100*relA* ^+^ Δ*dinJ-yafQ* were grown at 37°C for 24 hr in 96 well polystyrene plates in LB (A,B,C) or M9 (D,E,F) media. Attached cells were washed and planktonic cells were removed as described (Materials and methods). Dead cells stained with PI (red) and living cells retained staining with SYTO 9 (green). (A,D) Biofilms were photographed by CLSM with a using ×2.5, ×10 magnifications. The entire well was photographed from above. The image is a representative image from three independent experiments; each experiment was carried out in quadruplicate. Additionally, cells stained with SYTO 9 or PI were quantified as described in “materials and methods”. (B,E) Percentage of dead cells from the total population in the biofilm. (C,F) Absolute number of dead cells. Data were obtained from two independent experiments, performed quadruplicate.

## Supporting Information

S1 Fig LB: WT. (TIF)

S2 Fig LB: ΔmazEF. (TIF)

S3 Fig LB: ΔdinJyafQ. (TIF)

S4 Fig M9: WT. (TIF)

S5 Fig M9: ΔmazEF. (TIF)

S6 Fig M9: ΔdinJyafQ. (TIF)
