# Supplementary figures and images for: A Differential Effect of E. coli Toxin-Antitoxin Systems on Cell Death in Liquid Media and Biofilm Formation
Source: PLoS One. 2009 Aug 26;4(8):e6785. doi: 10.1371/journal.pone.0006785 (PMC2727947; doi:10.1371/journal.pone.0006785)

Fig. S1

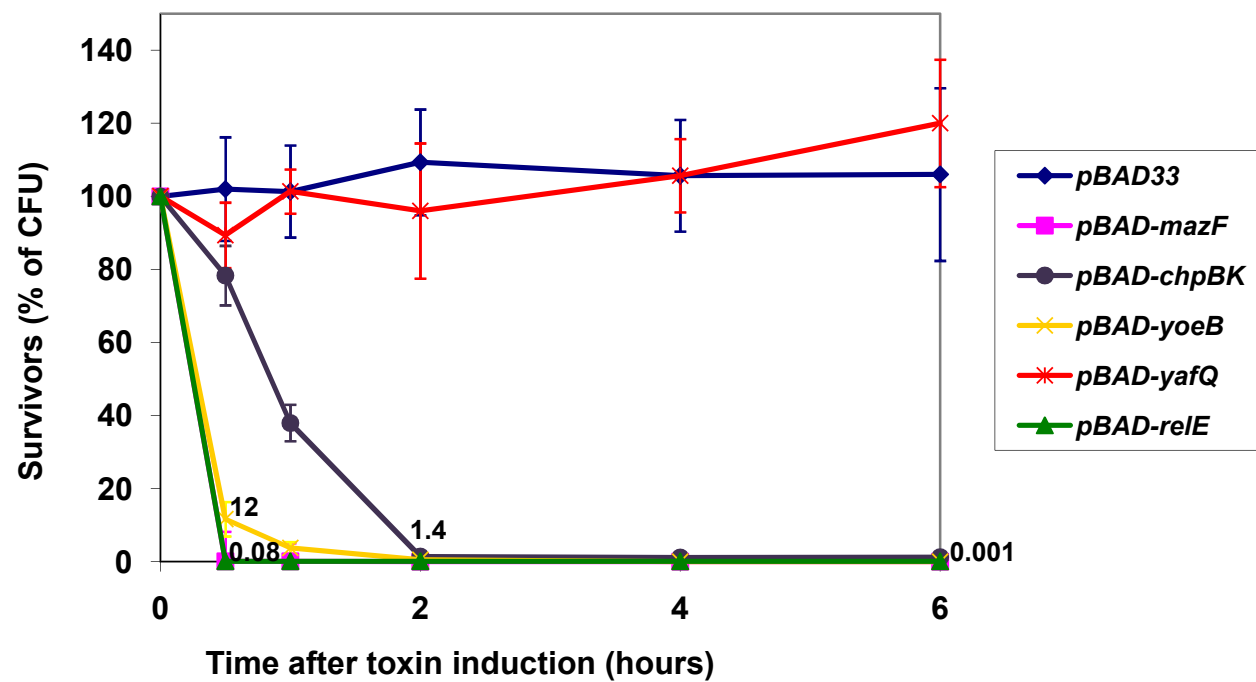

Supplement: Figure S1 — The effects of overproducing five E. coli toxins in cells grown in liquid M9 minimal medium. The E. coli strains used in the experiments from Fig. 1 were treated as described in the Legend to Fig. 1 except that cells were grown in M9-Glycerol medium (rather than in LB). (0.04 MB PDF) [file pone.0006785.s001.pdf]

Fig. S2

A. LB

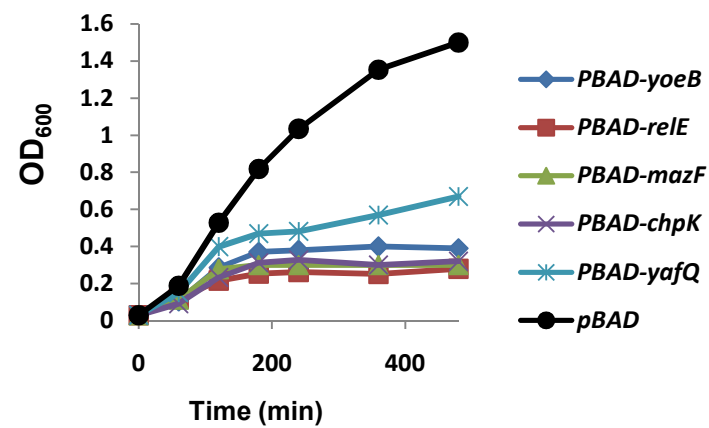

B. M9

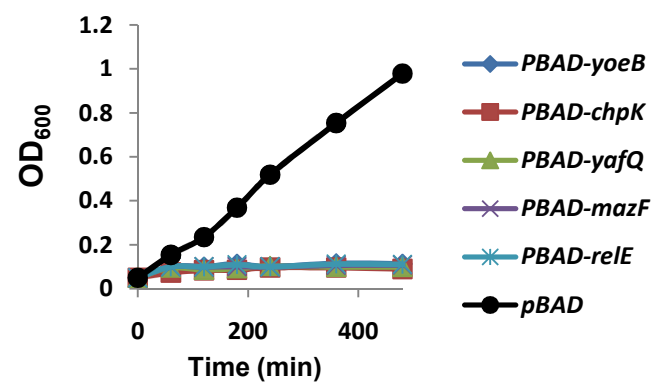

Supplement: Figure S2 — Growth arrest induced by the overproduction of five of E. coli chromosomally borne toxins. E. coli strain MC4100 was transformed with pBAD33 carrying an insert of one of five E. coli toxins. Cells were grown at 37°C in (A) liquid LB medium with 0.2% glucose (B) glycerol M9 minimal medium to an A600nm = 0.3–0.5. Then, growth medium of was changed into fresh medium with 0.2% arabinose. Bacterial growth was assessed by measuring A600 nm every 30 min. (0.05 MB PDF) [file pone.0006785.s002.pdf]

Fig S3

A. Rifampicin

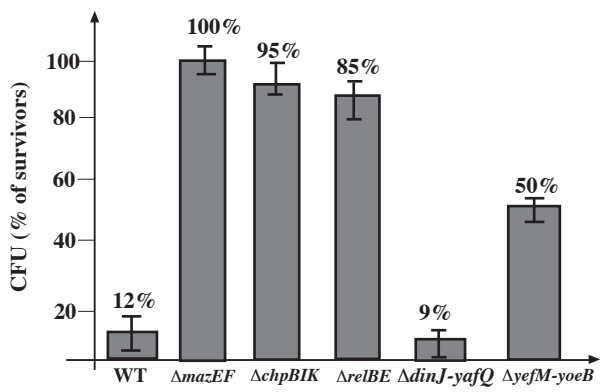

B. Spectinomycin

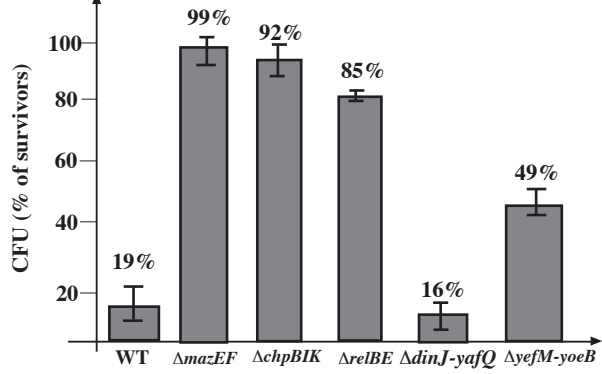

C. Trimethoprim

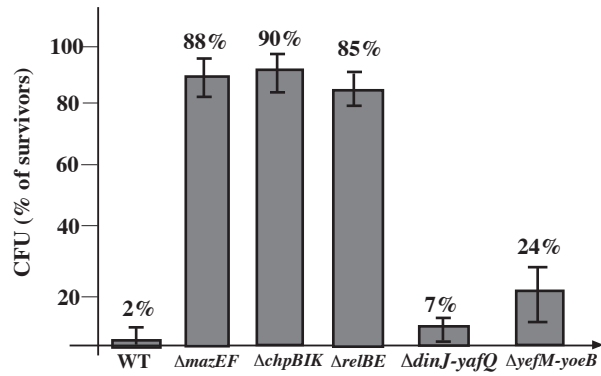

D. Nalidixic acid

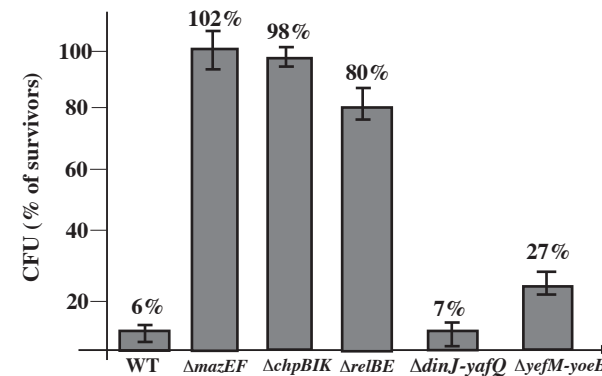

Supplement: Figure S3 — The effect of each of five of chromosomal encoded TA systems on E. coli cell survival following treatment with different antibiotics. E. coli strains: MC4100relA1 (WT) and its DchpBIK, DrelBE, DyefM-yoeB and DdinJ-yafQ derivatives (in gray) were grown to mid-log phase as described in Materials and Methods. Cells were incubated without shaking at 37°C with: (A) Rifampicin (25 µg/ml) for 10 min; (B) Spectinomycin (2 mg/ml) for 10 min; (C) Trimethoprim (2 µg/ml) for 2 hr; (D) Nalidixic acid (2 mg/ml) for 10 min. Cells were plated and CFUs assessed as described in Materials and Methods. (0.29 MB PDF) [file pone.0006785.s003.pdf]

**Fig S4**

**A. LB**

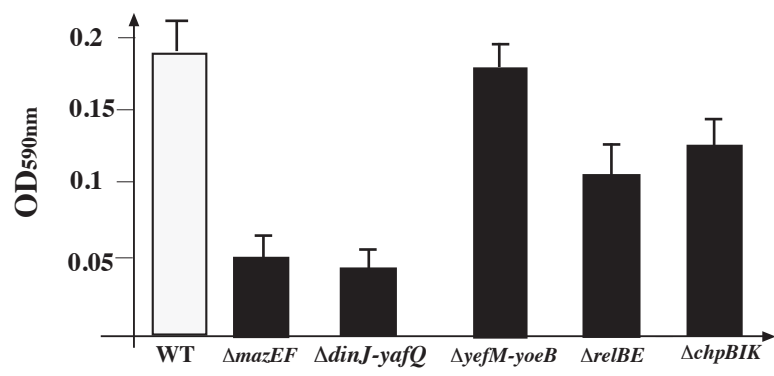

**B. M9**

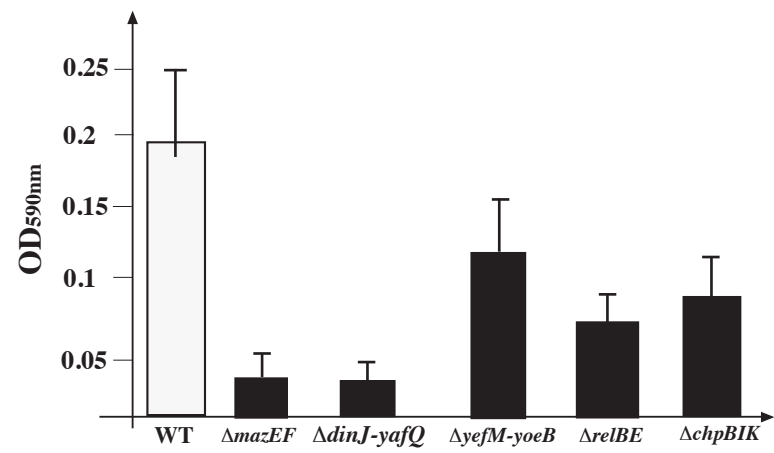

Supplement: Figure S4 — The effect of each of five of chromosomal encoded TA systems on early biofilm formation in E. coli. E. coli strains MC4100relA+ (WT) and its DmazEF, DchpBIK, DrelBE, DyefM-yoeB or DdinJ-yafQ derivatives were grown in 96 well polystyrene plates at 37°C for 8 hr in (A) LB or (B) M9. Quantification of CV-stained attached cells was done as described in Materials and Methods. (0.18 MB PDF) [file pone.0006785.s004.pdf]

Fig. S5

A. LB

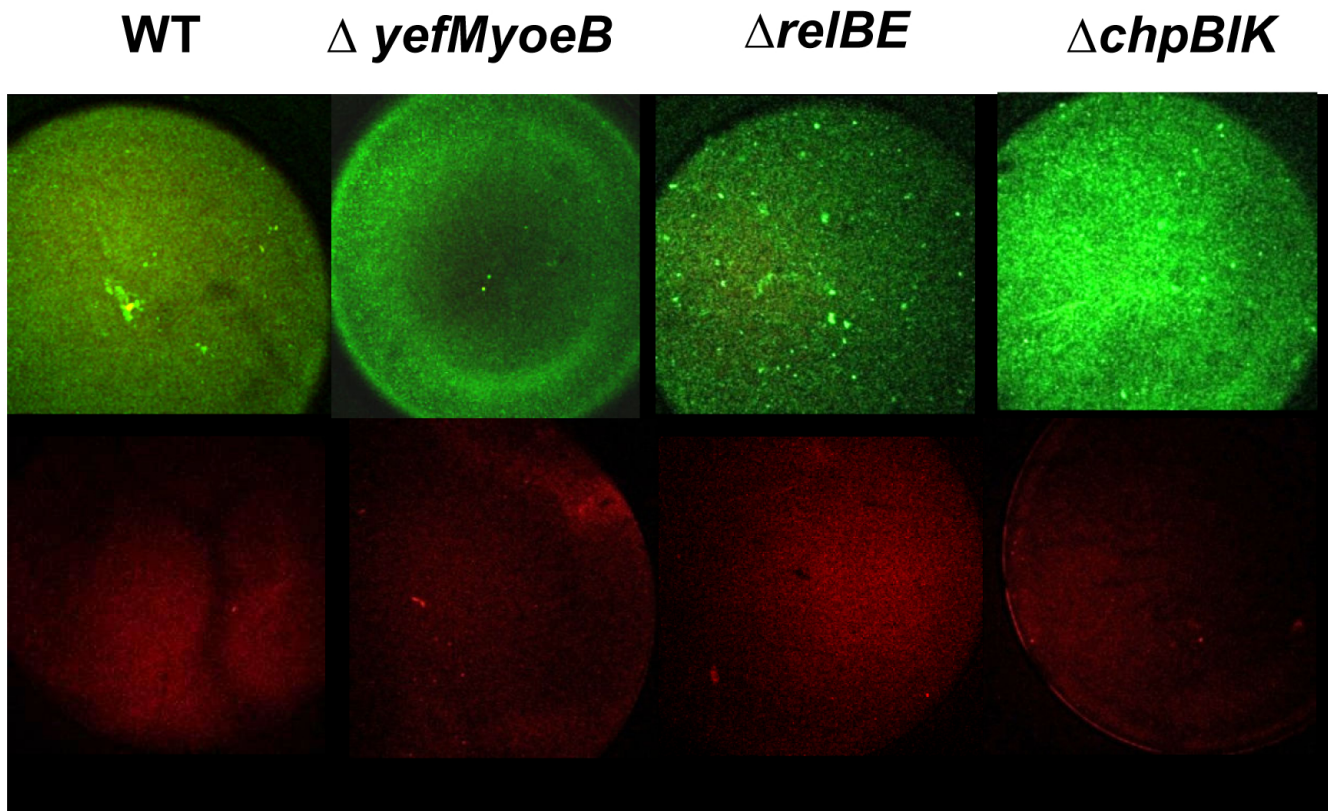

B.

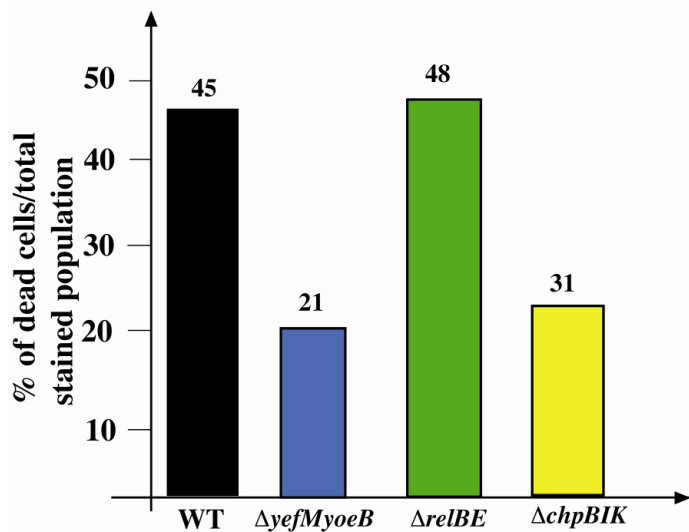

C.

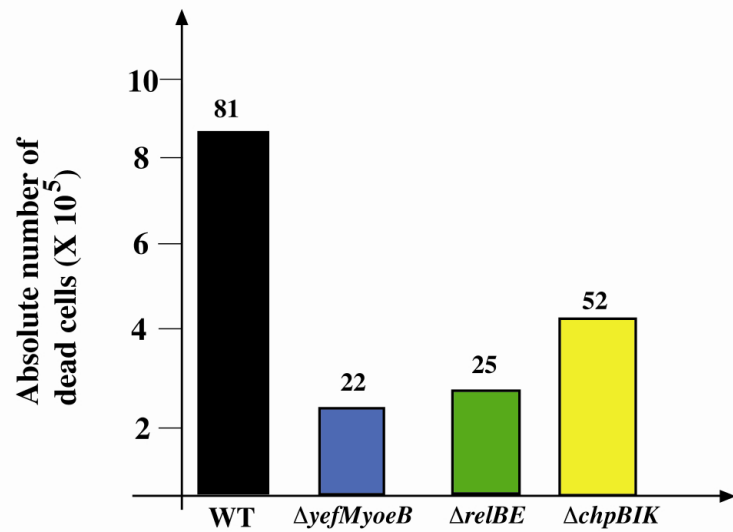

Supplement: Figure S5 — The effects of DchpBIK, DrelBE and DyefM-yoeB on E. coli cell death during biofilm formation in LB medium. E. coli strains: MC4100relA+ (WT) and its derivatives DchpBIK, DrelBE, and yefM-yoeB were grown in 96 wells polystyrene plates at 37°C for 24 hr in LB medium. For the rest of the experiment, see the Legend to Fig. 4. (6.81 MB PDF) [file pone.0006785.s005.pdf]

Fig. S6

A. M9

WT       $\Delta yefMyoeB$        $\Delta relBE$        $\Delta chpBIK$

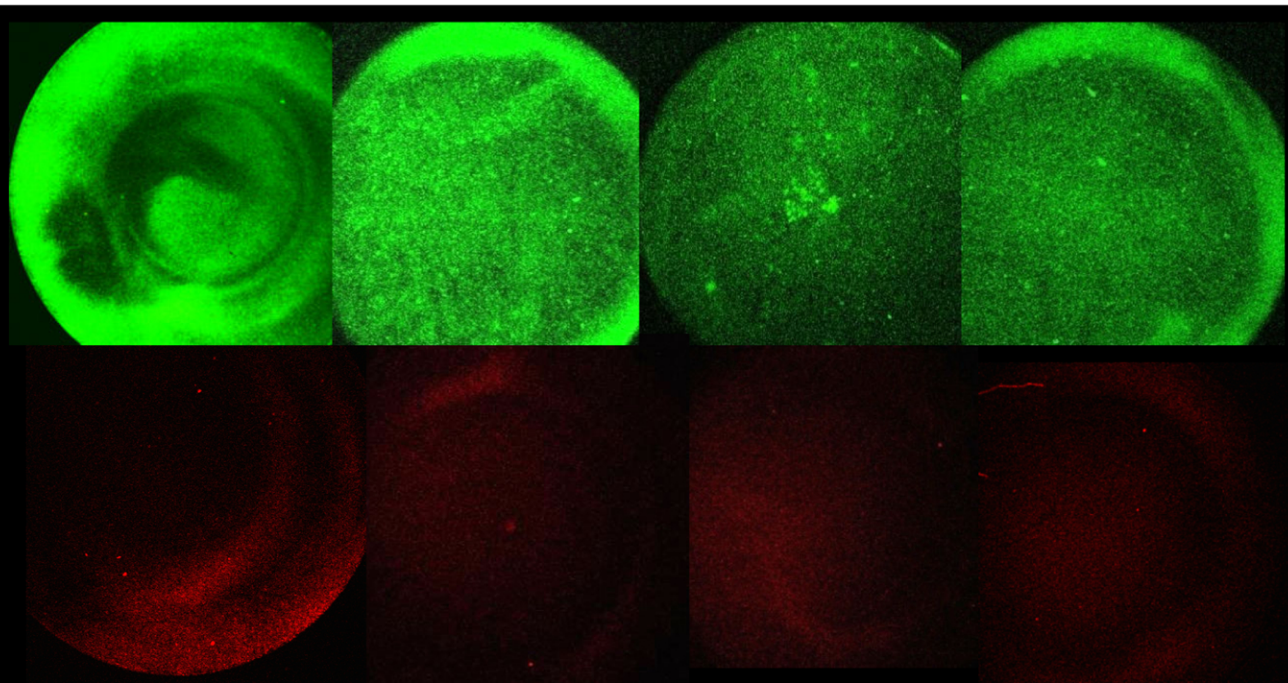

B.

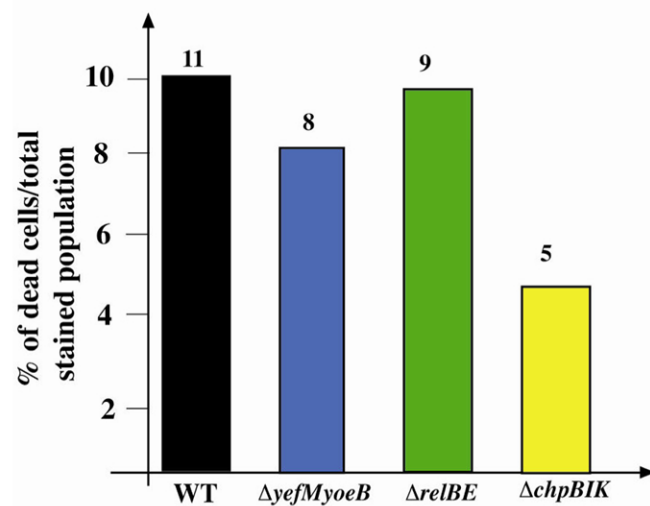

C.

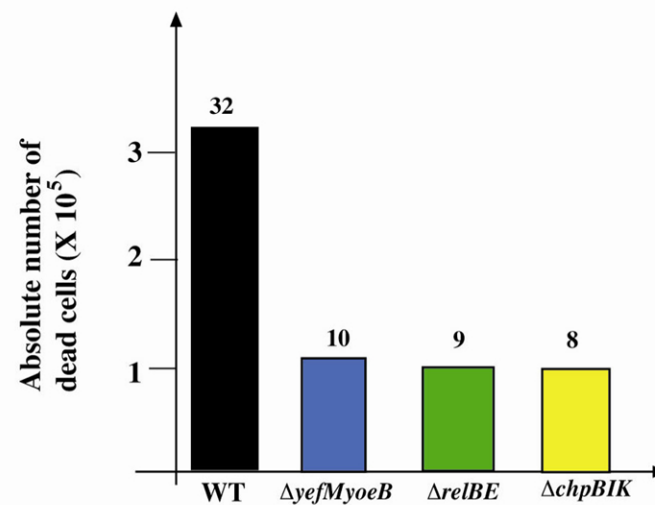

Supplement: Figure S6 — The effect of the deletions DchpBIK, DrelBE and DyefM-yoeB on E. coli cell death during biofilm formation in M9 medium. E. coli strains MC4100relA+ (WT) and its DchpBIK, DrelBE, and DyefM-yoeB derivatives were grown in 96 well polystyrene plates at 37°C for 24 hr in M9 medium. For the rest of the experiment, see the Legend to Fig. 4. (5.07 MB PDF) [file pone.0006785.s006.pdf]

Fig S7

A. 8h

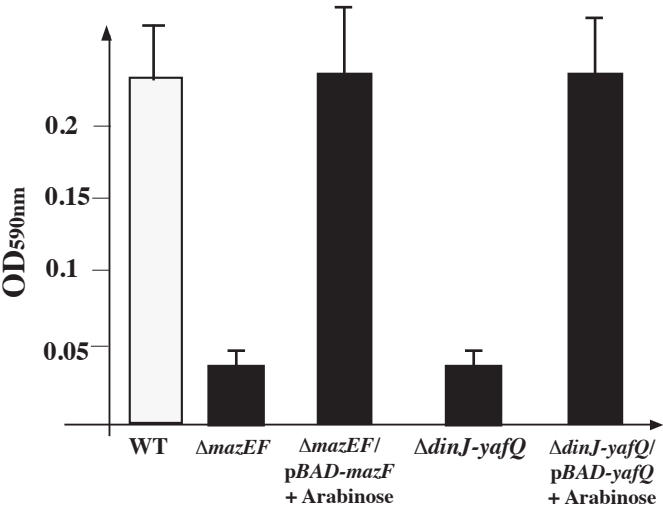

A. 24h

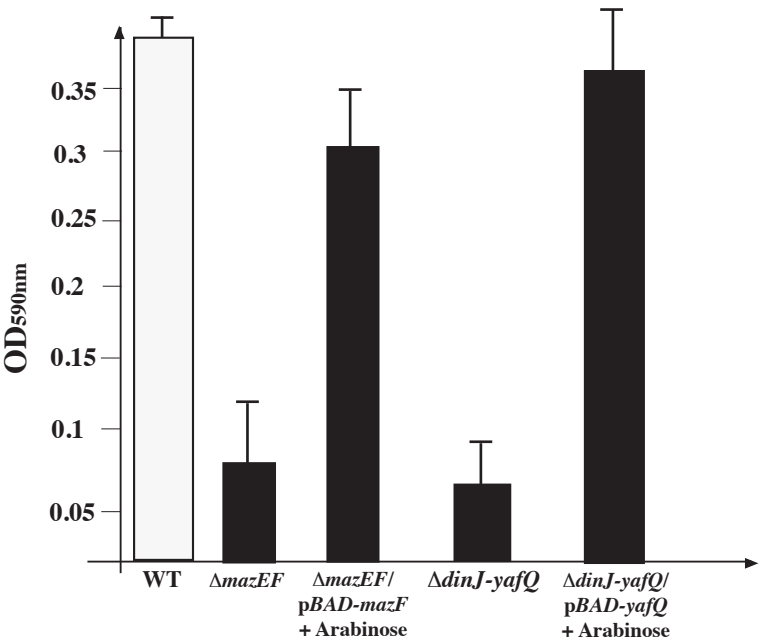

Supplement: Figure S7 — Overproduction of YafQ and MazF restores biofilm formation in a DmazEF and DdinJ-yafQ derivatives. E. coli strains MC4100relA+ (WT), MC4100relA+DmazEF/pBAD-mazF and MC4100relA+DdinJ-yafQ/pBAD-yafQ were grown in 96 wells polystyrene plates at 37°C in LB or LB+Arabinose 0.05% for (A) 8 h (B) 24 h. Quantification of CV-stained attached cells was done (Materials and Methods). (0.20 MB PDF) [file pone.0006785.s007.pdf]

Fig S8

A. LB

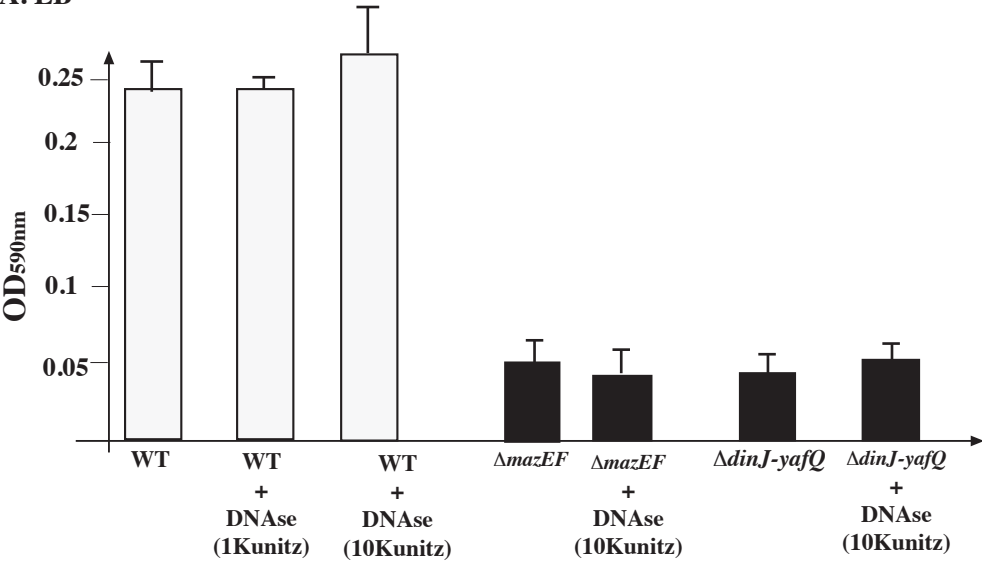

B. M9

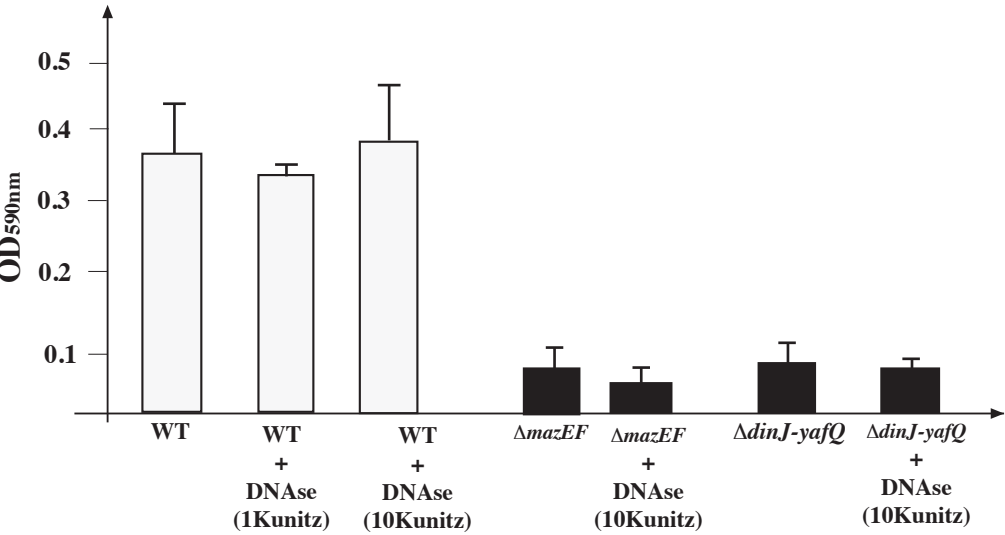

Supplement: Figure S8 — DNAse treatment does not affect early biofilm formation in E. coli. E. coli strains MC4100relA+ (WT) or its DmazEF and DdinJ-yafQ derivatives were grown in 96 wells polystyrene plates at 37°C for 8 h in (A)LB (B) M9. DNAse (1 or 10 Kunitz) was either applied or not applied to each well. Quantification of CV-stained attached cells was done (Materials and Methods). (0.21 MB PDF) [file pone.0006785.s008.pdf]

Fig S9

A. LB

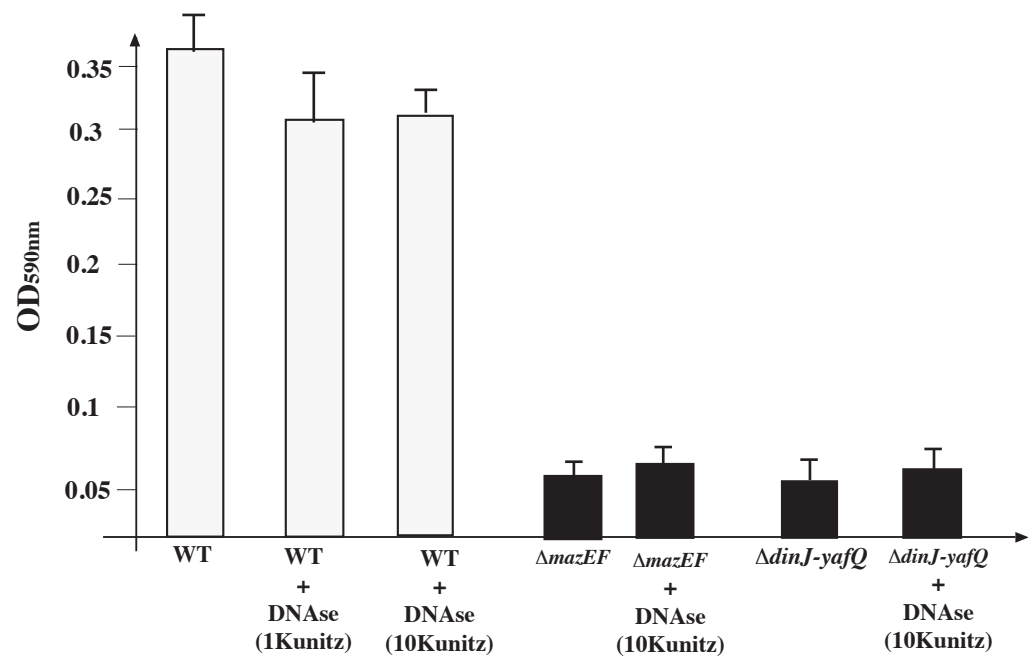

B. M9

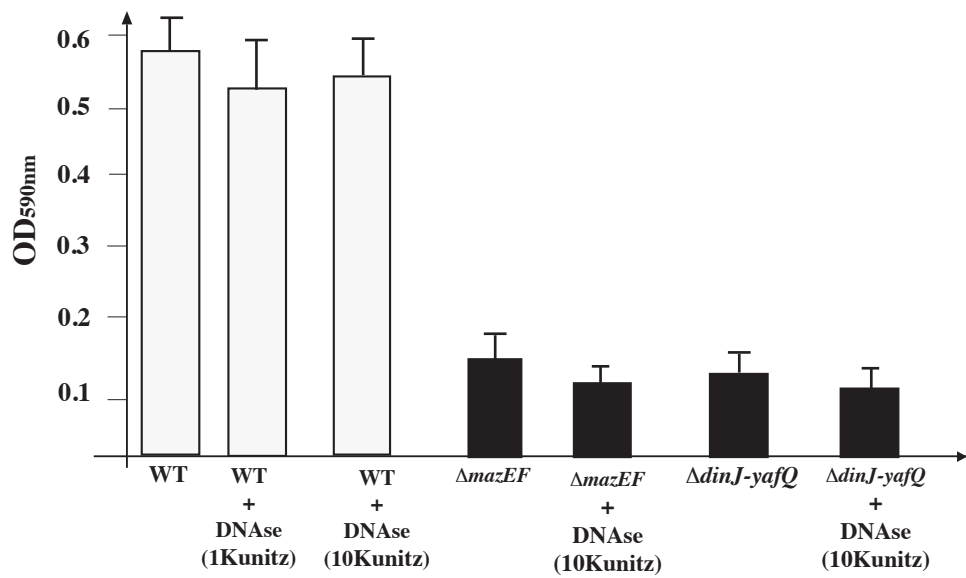

Supplement: Figure S9 — E. coli strains MC4100relA+ (WT) or its DmazEF and DdinJ-yafQ derivatives were grown in 96 wells polystyrene plates at 37°C for 24 h in (A)LB (B) M9. DNAse (1 or 10 Kunitz) was either applied or not applied to each well. Quantification of CV-stained attached cells was done (Materials and Methods). (0.22 MB PDF) [file pone.0006785.s009.pdf]
