# Supplementary figures and images for: Correction: A Differential Effect of E. coli Toxin-Antitoxin Systems on Cell Death in Liquid Media and Biofilm Formation
Source: PLoS One. 2015 Oct 2;10(10):e0140184. doi: 10.1371/journal.pone.0140184 (PMC4592190; doi:10.1371/journal.pone.0140184)

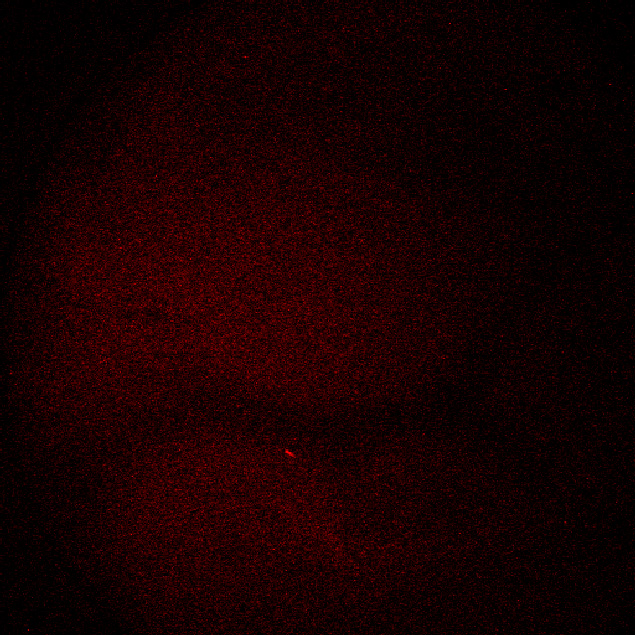

Supplement: S1 Fig — (TIF) [file pone.0140184.s001.tif]

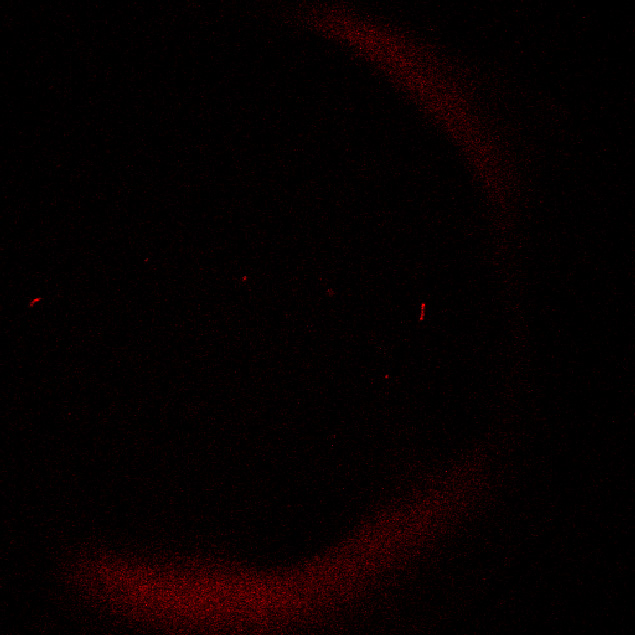

Supplement: S2 Fig — (TIF) [file pone.0140184.s002.tif]

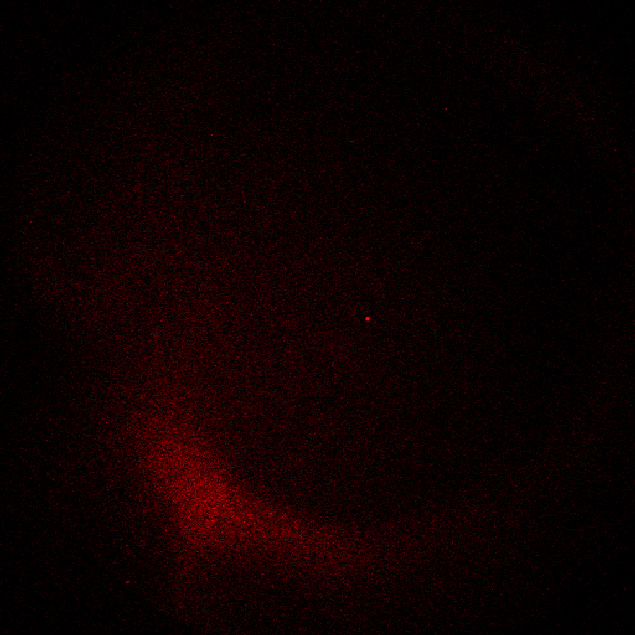

Supplement: S3 Fig — (TIF) [file pone.0140184.s003.tif]
